# Multimodal integration strategies for clinical application in oncology

**DOI:** 10.3389/fphar.2025.1609079

**Published:** 2025-08-20

**Authors:** Baoyi Zhang, Zhuoya Wan, Yige Luo, Xi Zhao, Josue Samayoa, Weilong Zhao, Si Wu

**Affiliations:** ^1^ AbbVie Bay Area, South San Francisco, CA, United States; ^2^ AbbVie, Inc., North Chicago, IL, United States

**Keywords:** deep learning, multimodal integration, oncology, prognosis, biomarker, treatment response

## Abstract

In clinical practice, a variety of techniques are employed to generate diverse data types for each cancer patient. These data types, spanning clinical, genomics, imaging, and other modalities, exhibit significant differences and possess distinct data structures. Therefore, most current analyses focus on a single data modality, limiting the potential of fully utilizing all available data and providing comprehensive insights. Artificial intelligence (AI) methods, adept at handling complex data structures, offer a powerful approach to efficiently integrate multimodal data. The insights derived from such models may ultimately expedite advancements in patient diagnosis, prognosis, and treatment responses. Here, we provide an overview of current advanced multimodal integration strategies and the related clinical potential in oncology field. We start from the key processing methods for single data modalities such as multi-omics, imaging data, and clinical notes. We then include diverse AI methods, covering traditional machine learning, representation learning, and vision language model, tailored to each distinct data modality. We further elaborate on popular multimodal integration strategies and discuss the related strength and weakness. Finally, we explore potential clinical applications including early detection/diagnosis, biomarker discovery, and prediction of clinical outcome. Additionally, we discuss ongoing challenges and outline potential future directions in the field.

## 1 Introduction

The rapid advancement of high-throughput technologies (Mardis, 2019), coupled with the digitalization of healthcare and electronic health records (EHRs) adoption (Jensen et al., 2012), has led to an unprecedented explosion of multi-modal datasets in oncology. These diverse data modalities include, but are not limited to, patient clinical records, multi-omics data—spanning genomics, transcriptomics, proteomics, and metabolomics—at bulk, single-cell, and spatial levels, as well as medical imaging (magnetic resonance imaging [MRI], computed tomography [CT], histopathology) and wearable sensor data. Each of these modalities provides unique insights into cancer diagnosis (Carrillo-Perez et al., 2022; Cui C. et al., 2022), prognosis (Lobato-Delgado et al., 2022; Zhu et al., 2020), and treatment responses (Chen Z. et al., 2024; Keyl et al., 2025; Wang et al., 2023), yet their true potential lies in their integration (Boehm et al., 2022; Unger and Kather, 2024). Multi-modal data fusion enables the combination of orthogonal information, allowing different data types to complement one another and augment the overall information content beyond what a single modality can provide (Kline et al., 2022; Miotto et al., 2018). By integrating these diverse datasets, researchers can achieve a more comprehensive understanding of complex biological processes, improve inference accuracy, and enhance clinical decision-making, ultimately driving advancements in precision oncology.

Although multi-modal integration holds great promise for improving disease modeling and biomarker prediction, it presents several challenges due to the scale, complexity, and heterogeneity of the data. A primary challenge is data heterogeneity (Li et al., 2022a; Tang Z. et al., 2024; Zhang et al., 2025), as different modalities often vary in format, structure, and coding standards, and may originate from multiple vendors or institutions, making normalization and harmonization crucial before integration. Additionally, data quality and completeness issues, such as missing values, inconsistencies, and noise, can compromise both integration efforts and model performance (Cui et al., 2022; Waqas et al., 2024; Zhao et al., 2024). The computational and storage demands of large-scale multi-modal datasets—particularly high-resolution imaging and raw genomics data—necessitate advanced infrastructure and scalable analytical tools to enable efficient data (pre)processing and integration. Furthermore, multi-modal fusion methods have evolved in diverse directions, yet standardized methodologies and workflows remain underdeveloped (Chen et al., 2023; Chen et al., 2021a). Addressing these challenges requires comprehensive data frameworks encompassing preprocessing, alignment, harmonization, and integration, along with improved storage solutions, computational resources, and interdisciplinary collaboration. Overcoming these barriers is key to unlocking the full potential of multi-modal data for precision medicine and clinical decision-making.

Over the past decades, artificial intelligence (AI) technologies have grown rapidly and demonstrated immense potential in clinically relevant tasks. They excel at handling complex datasets and extracting meaningful clinical insights—capabilities that typically require years of human training and experience. For instance, image-based models have been developed to assist in cancer diagnosis, staging, grading and subtyping by analyzing morphological features in histopathological images (Araújo et al., 2017; Arvaniti et al., 2018; Coudray et al., 2018; Kanavati et al., 2021; Nagpal et al., 2020; Wan et al., 2017). Large language models have been applied to transform unstructured clinical notes into structured data, facilitating centralized data strategy and enabling more efficient downstream analysis (Luo et al., 2022; Sorin et al., 2024; Van Veen et al., 2024). Moreover, AI is particularly well-suited for integrating diverse data modalities. Various deep learning models have been developed to infer genomic data from imaging data (Diao et al., 2021; El et al., 2024; Jané et al., 2023; Jin et al., 2024; Kather et al., 2020; Shamai et al., 2022; Zhang et al., 2024a). Therefore, AI-driven multimodal data integration can benefit multiple aspects of clinical development, including biomarker discovery, patient stratification, and clinical trial recruitment.

Although several reviews on AI methods for multimodal integration have been published in past years (Boehm et al., 2022; Waqas et al., 2024; Kline et al., 2022; Stahlschmidt et al., 2022), most have not detailed the technical aspects underlining these strategies. Additionally, discussions on emerging technologies such as spatial transcriptomics, single-cell sequencing, and most-recent advanced computational methods were not discussed due to the publication dates of those reviews. Thus, this review explores the technical specifics of both established and newly developed methods, while incorporating discussions on novel data types. We specifically highlight key AI approaches for integrating common data modalities and explore their potential applications in clinically relevant tasks. In the last section, we discuss existing challenges and future directions for advancing AI-driven multimodal data integration.

## 2 Unimodal processing

### 2.1 Omics

#### 2.1.1 Traditional omics data

In the context of traditional omics data, unimodal processing refers to the independent analysis of a single data type before any cross-modality integration. This approach ensures rigorous quality control, effective noise reduction, and precise feature extraction. For instance, genomic analysis employs tools such as GATK (Valle et al., 2016), MuTect (Aaltonen et al., 2020), and VarScan (Koboldt et al., 2012) to detect mutations and structural variants—as demonstrated in the TCGA pan-cancer study that utilized whole-genome sequencing to identify critical mutations across various cancer types. Transcriptomic analysis uses methods like DESeq2 and EdgeR to quantify gene expression and determine differential expression patterns associated with distinct disease subtypes (Siavoshi et al., 2022; Varet et al., 2016).

To further simplify the complexity of high-dimensional gene expression data, dimension reduction techniques such as principal component analysis (PCA) capture the primary axes of variation, effectively summarizing the most variable transcriptional patterns across samples (Alter et al., 2000). These components can simplify complex datasets and have been used to identify molecular subtypes of cancer with distinct biological behaviors and prognostic implications (Perou et al., 2000). Additionally, gene signatures are predefined sets of genes whose collective expression patterns are associated with specific biological states or clinical phenotypes (Subramanian et al., 2005). They have been extensively used in current clinical workflow. For example, the PAM50 gene signature is widely used for classifying breast cancer into intrinsic subtypes with prognostic relevance (Parker et al., 2023). Systematic identification of gene signatures can improve patient stratification by correlating molecular features to disease progression and treatment response, as demonstrated by the use of Oncotype DX to assess breast cancer recurrence risk (Liberzon et al., 2015). Pathway-based approaches involve mapping significant gene expression changes onto known biological pathways, such as those cataloged in KEGG or Reactome databases (Kanehisa and Goto, 2000). These methods can reveal dysregulated cellular processes and signaling networks in cancer, providing functional insights into disease mechanisms and potential therapeutic targets, exemplified by a colorectal cancer study using KEGG pathway enrichment analysis (Croft et al., 2011). Pathway analysis has been especially useful for interpreting high-dimensional transcriptomic data and linking molecular alterations to broader biological functions (Khatri et al., 2012).

#### 2.1.2 Single-cell and spatial omics data

Recent advances in single-cell and spatial sequencing technologies have revolutionized cancer research by enabling high-resolution analyses of tumor biology (Zhang Y. et al., 2021; Lewis et al., 2021), revealing cellular heterogeneity and spatial organization within tumor microenvironment. While most of the related methods are still predominantly utilized in research settings, they offer a more detailed surrogate of the tumor profile, which cannot be achieved through bulk sequencing techniques. But these emerging technologies are increasingly poised to be applied in clinical settings.

Single-cell RNA sequencing (scRNA-seq) facilitates gene expression profiling at the individual cell level, uncovering rare cell populations, diverse cellular states, and dynamic transcriptional changes that bulk sequencing approaches might obscure (Macosko et al., 2015). This technique has been instrumental in characterizing tumor cell plasticity, immune infiltration, and resistance mechanisms across various cancers, such as the discovery of intra-tumoral heterogeneity in triple-negative breast cancer (Patel et al., 2014).

Spatial transcriptomics complements single-cell data by preserving the spatial context of gene expression. Platforms such as 10x Genomics Visium, Xenium, Nanostring GeoMx, CosMx, Slide-seq, and image mass cytometry enable the simultaneous measurement of gene expression and histological features, facilitating the mapping of transcriptional patterns to specific tissue regions (Ståhl et al., 2016; Nagasawa et al., 2024). Spatial transcriptomics has been instrumental in studying the tumor-immune microenvironment across various cancer types—including colorectal and gastric cancers, melanoma, breast cancer, non-small cell lung cancer, pancreatic cancer, and glioblastoma—where cellular interactions and tumor architecture are crucial for understanding disease progression. (Du et al., 2023; Nagasawa et al., 2024; Zhu et al., 2024; Boe et al., 2024; Lewis et al., 2021).

Moreover, combining traditional bulk omics data with single-cell and spatial omics can offer a more comprehensive view of tumor biology, supporting better-informed clinical decisions and personalized treatment strategies. By integrating these modalities, researchers can capture both the overall molecular landscape and the intricate details of cellular heterogeneity and spatial organization, uncovering nuances that are often masked in bulk analyses. This approach has been successfully demonstrated in studies combining TCGA data with single-cell approaches in melanoma (Tirosh et al., 2016) and ovarian cancer (Lähnemann et al., 2020).

In terms of AI application in single-cell and spatial transcriptomics data, several foundational models have been developed using extensive single-cell datasets, including scVI, scBERT, Geneformer, and scGPT (Lopez et al., 2018; Yang F. et al., 2022; Theodoris et al., 2023; Cui et al., 2024). These models have shown superior performance in tasks like cell type annotation and correcting batch effects (Kedzierska et al., 2025; Boiarsky et al., 2024). For spatial transcriptomics, generative models are broadly utilized to map spatial transcriptomics into latent space embeddings for more effective representation than the gene expression matrix (Chang et al., 2022; Long et al., 2023; Xu et al., 2024b). Clustering these derived embeddings helps identify distinct spatial domains from whole slide images. To identify spatially variable genes with expression patterns changing gradually across slides, Gaussian process models and Markov random fields are employed (Svensson et al., 2018; Sun et al., 2020; BinTayyash et al., 2025; Zhang et al., 2022). Additionally, graph neural networks are used to model cellular neighborhoods *via* graph structures, enabling the quantification of cell-cell interactions between different cell types (Yuan and Bar-Joseph, 2020; Fischer et al., 2023).

### 2.2 Images

#### 2.2.1 Histology images

Histology images of stained tissue sections are central to cancer diagnosis: biopsy samples are fixed and stained (e.g., hematoxylin and eosin [H&E]) and examined by pathologists to assess malignancy, subtype, grade, and other histopathological features. Despite the routine digitization of whole-slide images (WSIs), these gigapixel datasets remain underutilized relative to genomic and other omics resources (Saltz et al., 2018). This landscape is rapidly changing - advances in deep learning (DL) models carry the promise to extract prognostic and predictive signals with clinical relevance directly from H&E slides (Saltz et al., 2018; Dang et al., 2025; Shamai et al., 2022). Indeed, several models have been proposed to aid pathologist to locate potential tumor tissue from H&E slides. Some of them (e.g., Paige Prostate (Rienda et al., 2024; Introducing FDA-Approved Paige Prostate, 2025) and Roche Digital Pathology Dx (“Roche Receives FDA Clearance on Its Digital Pathology Solution for Diagnostic Use, 2025)) also received FDA Breakthrough Device Designation. However, whole-slide images present unique bioinformatic challenges: a single WSI may yield hundreds of thousands of tiles, each exhibiting staining variability and often lacking region-level annotations (Gadermayr and Tschuchnig, 2024). To address these issues, contemporary pipelines first learn tile-level feature embeddings—using either supervised or self-supervised approaches—and then aggregate these embeddings into slide-level predictions *via* pooling or attention-based mechanisms (Xu et al., 2024a), as detailed in the following sections.

##### 2.2.1.1 Fully-supervised learning

Fully supervised learning has pioneered the analysis of medical images in oncology, enabling tasks such as tumor detection and classification by training models on meticulously labeled datasets (Otálora et al., 2021; Turkki et al., 2015). In general, this class of method excels in utilizing annotated data to achieve good specificity and sensitivity. However, when dealing with commonly data models in histopathology, such as H&E stained images, fully supervised learning presents significant challenges (Lu et al., 2022; Rodriguez et al., 2022). Each input tile extracted from the source WSI requires explicit labeling, a process that is both labor-intensive and at times impractical. This requirement for fine-grained annotation restricts the availability and diversity of trainable datasets, ultimately limiting model generalizability across varied pathological contexts. Such limitations emphasize the emergence of self-supervised learning (SSL) methods as a superior alternative, offering the ability to harness vast amounts of unlabeled data, to reduce dependency on exhaustive manual annotation, and to enhance the robustness and adaptability of models in digital pathology.

##### 2.2.1.2 Self-supervised learning methods

Recent advances in SSL have introduced contrastive methods that effectively distinguish between positive and negative data pairs. These methods generate multiple augmented views of an image and use encoding techniques to enhance the similarity between related pairs while differentiating them from unrelated ones (Gui et al., 2024; Kang et al., 2023). Prominent examples such as SimCLR (Chen T. et al., 2020) and MoCo (Chen and He, 2021; He K. et al., 2020) achieve performance similar to fully-supervised methods without relying on labeled data, and have been successfully applied to pathological image processing (Ciga et al., 2022; Gadermayr and Tschuchnig, 2024). These methods are praised for their conceptual simplicity and modular design, often employing unique data augmentation techniques and a multilayer perceptron (MLP) as a projection head. However, a notable drawback is high computational demands. For SimCLR, large batch sizes are needed to provide diverse negative pairs, leading to high memory costs. To address these, implementations often approximate the loss by reducing the number of comparisons to random subsets during training (Chen et al., 2020). Another mitigation design can be found in MoCo, which features a mechanism for maintaining a dynamic dictionary of negative samples (He K. et al., 2020). This approach allows for the efficient use of extensive dictionaries without the necessity for large batch sizes, a feature that has been further refined in MoCo v2 (Chen and He, 2021).

Subsequently, non-contrastive methods have emerged with a significant improvement in operational efficiency. Typical methods of this class achieve learning by utilizing two neural networks, often configured as Siamese networks (Bromley et al., 1993), in an asymmetrical (Chen and He, 2021; Grill et al., 2020) or symmetrical model architecture (Zbontar et al., 2021). Unlike contrastive SSL methods, non-contrastive approaches focus exclusively on aligning positive view pairs augmented from the same image, also earning the designation “self-distilled.” (Gui et al., 2024). With increasingly light-weight designs, notable examples of this method family further close the performance gap with, sometimes even exceed, their supervised learning benchmarks (Zbontar et al., 2021; Grill et al., 2020). Common challenges include trivial solutions, i.e., model weights collapsing to a constant during training. Therefore, popular implementations of non-contrastive methods primarily differ in their ways to avoid such trivial solutions, as discussed below.

BYOL (Bootstrap Your Own Latent) is the first method of this kind (Grill et al., 2020). This method employs two networks termed online and target networks. The online network consists of an encoder, a projector, and a predictor; while the target network, sharing the architecture of the online network, uses parameters which are a moving average of the online network’s parameters. This design facilitates the online network’s learning through self-supervised means, avoiding collapsing solutions through its momentum encoder. On the other hand, SimSiam (Simple Siamese Network) utilizes a pair of identical networks that share weights but apply stop-gradients to prevent trivial solutions (Chen and He, 2021). The constraint of dependency on asymmetric network design to prevent collapse in BYOL and SimSiam was relaxed by a more recent method, Barlow Twins (Zbontar et al., 2021). Inspired from neuroscientist Barlow (Barlow, 2012), this method applies redundancy reduction principles through identical networks. The core idea of avoiding collapsing solutions is to compute the cross-correlation matrix between outputs of the networks fed with distorted versions of an input, aligning this matrix as closely as possible to an identity matrix (Zbontar et al., 2021). It was pointed out, however, the Barlow Twins method can be sensitive to certain data augmentations, a trait shared with SimCLR but not observed in BYOL.

Clustering-based SSL methods offer a distinct approach by leveraging clustering algorithms to group data into meaningful representations. In general, these methods assign different views of input data to clusters and train the model to differentiate between clusters instead of individual representations, as seen in contrastive methods (Gui et al., 2024). By clustering features from unlabeled data, clustering methods allow models to understand the data distribution, obviating the need of large negative samples. For instance, DeepCluster (Caron et al., 2018) iterates between clustering image descriptors and updating network weights using cluster assignments as pseudo-labels, refining its learning in an end-to-end manner. As one of the founding SSL methods, DeepCluster is flexible with clustering algorithms like K-means, and supports various architectures [e.g., AlexNet, VGG (Krizhevsky, Sutskever, and Hinton, 2012; Simonyan and Zisserman, 2015)] to enhance feature transfer performance. Nevertheless, issues like empty clusters still haunt in early clustering methods; and their struggles with large datasets due to requiring full dataset passes for updates further limit their real-world applications. SwAW, a successor to DeepCluster, offers improvements by reformulating clustering-based SSL as an online method (Caron et al., 2020). Together with its novel multi-crop data augmentation strategy, SwAV enables scalable learning with smaller memory and computational requirements. Notably, SwAV shares the model designs of contrastive SSL by having “swapped” predictions, where the model predicts the cluster assignment of one view from the representation of another view. Despite reducing computational demands, it has been pointed out that SwAV has to carefully incorporate symmetry-breaking mechanisms to prevent trivial solutions, especially in scenarios with a high ratio of clusters to batch size (Zbontar et al., 2021).

Self-distillation methods leverage the concept of knowledge distillation (Hinton et al., 2015) for SSL. Knowledge distillation involves two models: a teacher model and a student model. The teacher model is typically large, complex, and extensively pre-trained on large datasets, while the student model is smaller and simpler. In the training process, the student model is trained to learn the output distribution (soft labels) of the teacher model instead of categorical classes (hard labels). This allows the student model to learn the rich representations encoded by the teacher model, enhancing its performance. In terms of self-distillation, there are two typical methods: Distillation with No Labels (DINO) (Caron et al., 2021) and Image BERT Pre-Training with Online Tokenizer (iBOT) (Zhou et al., 2022). DINO generates multiple crops of each image as different views. The original image is processed through the teacher model to learn a global representation, while the crops are processed through the student model to learn local representations. The objective is to minimize the distance between global and local representations since they are from the same original image. iBOT were developed based on masked image modeling. During training, the student model learns to reconstruct masked crops under supervision of teacher models on same but unmasked crops.

A significant advantage of SSL is that it does not require labeled data, allowing for the full utilization of available datasets. As a result, researchers began to develop large models using extensive datasets curated from both public and proprietary sources, the so-called foundation models (Alfasly et al., 2024). Foundation models leverage SSL pretraining on massive amounts of data, enabling them to learn richer representations from input data, and achieve superior and more robust performance compared to fully-supervised models. Besides image-only SSL methods, some foundation models, inspired by vision-language models (Bordes et al., 2024), also take image-text pairs consisting of pathological images and corresponding captions as input (Huang et al., 2023; Ikezogwo et al., 2025; Lu et al., 2024a).

##### 2.2.1.3 Slide-level aggregation

Since each whole slide image may contain a large number of tiles, it is essential to aggregate tile-level embeddings together for slide- or patient-level predictions, the so-called multi-instance learning (MIL) (Gadermayr and Tschuchnig, 2024). MIL focuses on predicting the label of a set of instances without knowing the label of each individual instance. Naïve methods such as averaging tile-level embeddings or predictions into slide-level embeddings or predictions, have been employed (Coudray et al., 2018; Kather et al., 2019). However, this approach treats all tiles equally and overlooks the fact that different tiles may exhibit distinct morphological features, leading to varying contributions to the prediction task. To address this, prior research has incorporated modules to identify the most important tiles and utilized only these top tiles for predictions (Campanella et al., 2019; Campanella et al., 2018; Courtiol et al., 2019). To leverage features from all tiles, attention-based methods have been developed, where an attention module is added to assign an attention score to each tile based on its embedding, enabling a weighted summation of all the tile embeddings (Carmichael et al., 2022; Ilse et al., 2018; Li et al., 2021b; Schirris et al., 2022).

With the recent advancements in transformer architecture, the self-attention (Vaswani et al., 2023) mechanism has been introduced to enhance the original attention mechanism (Li et al., 2021a; Shao et al., 2021; Zhao et al., 2022). The advantage of self-attention is that it accounts for pairwise correlations between tile-level embeddings, assigning attention to each tile in the context of all tiles rather than based on a single tile embedding. Furthermore, self-attention incorporates the spatial relationship of tiles through position encoding methods, allowing position information of all tiles to be encoded and passed to the model (Li et al., 2021a; Shao et al., 2021; Zhao et al., 2022). Besides self-attention, graph structures have also been used to capture spatial proximity between tiles (Chen et al., 2021b; Li et al., 2018). In this approach, a whole slide image is represented as a graph, where nodes represent tiles and edges reflect direct proximity between two tiles. Graph neural networks can then be used to aggregate node-level (tile-level) embeddings into a whole graph embedding (slide-level). Further research has also explored the application of self-attention mechanisms within graph structures (Ding S. et al., 2023; Zheng et al., 2022).

In addition, cluster-based methods have been employed to aggregate tile-level embeddings into slide-level embeddings (Lu et al., 2021; Yao et al., 2020; Yao et al., 2019). These methods first assign all tiles from a slide into several morphology-related clusters through unsupervised methods to reduce dimensionality. Next, they extract cluster-level embeddings for each cluster and aggregate all cluster-level embeddings into a slide-level embedding. This aggregation can be completed through simple concatenation or attention-based summation.

While most MIL methods are weakly-supervised as previously described, some studies have explored self-supervised MIL approaches to obtain slide-level embeddings without any labels. To achieve this, researchers extended the Vision Transformer (ViT) (Dosovitskiy et al., 2021) architecture to WSIs. Specifically, ViT processes an image of 256 × 256 pixels by cropping it into non-overlapping 16 × 16 patches. Then each patch is tokenized, and self-attention is calculated between tokens to derive the embedding of the original image. However, applying ViT to WSIs substantially increases computational costs due to the gigapixel size of WSIs, which can result in enormous tiles and make self-attention calculation impossible. To reduce computational costs, the Hierarchical Image Pyramid Transformer (HIPT) has been proposed (Chen et al., 2022a). HIPT breaks down the patching process into hierarchical levels, where the model first learns embeddings from small tiles, then progressively learns from larger tiles composed of these small tiles, ultimately learns the embeddings for the entire WSI. Prov-GigaPath (Xu et al., 2024a) leveraged the dilated attention (Ding J. et al., 2023) to replace the vanilla attention, reducing the computational complexity from quadratic to linear and enabling the attention calculations for billions of tokens (tiles). Since data labeling can be often costly and challenging to procure, these slide-level self-supervised learning techniques offer promising avenues for future research.

#### 2.2.2 Radiology images

Unlike histopathology images, which requires tissue extraction, radiology imaging is a non-invasive technique that enables lesion detection on a tissue-wide scale and aids in clinical decision-making. CT and MRI are the two most prevalent data modalities within radiology. In terms of data structure, radiology images differ from histopathology images in following ways. Typically, radiology images are three dimensional, composed of several stacked 2D slices scanned at various locations within the human body, while histopathology images are usually 2D. Radiology images are smaller, with thousands of pixels per edge, whereas histopathology images usually contain gigapixels. Despite these differences, radiology images share several similar processing techniques with histopathology images. Initially, research focused on extracting hand-crafted features (e.g., lesion size) for clinical applications (Sousa et al., 2010; Riccardi et al., 2011; Ye et al., 2009). With advancements in deep learning, widely applied image-based deep learning models such as convolutional neural networks (CNNs) (Katzman et al., 2018; Wang et al., 2022a) and vision transformers (Murphy et al., 2022; Wollek et al., 2023) have been used to learn representative features from radiology images. Similar to histopathology, multi-instance learning approaches have been employed to integrate different radiology slices instead of tiles from whole slide images (Shin et al., 2019; Zhang et al., 2020). Several studies have developed foundation models for radiology, showing promising performance in clinically relevant tasks such as nodule classification and survival prediction (Pai et al., 2024; Paschali et al., 2025; Wu et al., 2023). In terms of clinical application, radiomics-based AI models account for the highest proportion (∼70%) of FDA approved AI tools till 2023, with common use cases including image reconstruction, tissue segmentation, and abnormal tissue detection (Joshi et al., 2024; Luchini et al., 2022).

### 2.3 Electronic health records

Electronic health records (EHRs) contain patient clinical information in both structured and unstructured data formats. Structured data is typically organized in tabular formats that include features such as diagnosis codes, laboratory test results, and lines of therapy for each entry. In contrast, unstructured data is more complex and often consists of clinical notes. Traditional machine learning methods, such as regression-based and kernel-based methods, have been employed to analyze structured data by correlating it with clinical outcomes (Daemen et al., 2012; Jarvis et al., 2013). More recently, neural networks have been widely used in embedding structured data into compact vector spaces to enhance predictive capabilities for clinical outcomes (Keyl et al., 2025; Rasmy et al., 2018; Shickel et al., 2018). For unstructured data, natural language processing (NLP) tools have been extensively used either to extract important information from clinical notes, converting them into structured data (Gholipour et al., 2023; Kehl et al., 2020), or to directly embed entire clinical notes into highly compact vector spaces for downstream predictions (Lu et al., 2024a; Xiang et al., 2025). Several companies have already integrated NLP tools into their clinical workflows to streamline data curation and enhance efficiency (Swaminathan et al., 2020). Taking together, we summarized above discussed preprocessing methods across different modalities in Table 1.

**TABLE 1:** Summary of preprocessing methods across different modalities.

Preprocessing methods	Modality	Special user case
PCA, Gene signature, Pathway analysis	Omics	Feature extraction from multi-omics data
Single cell foundation model	Omics	Learning a better representation from single cell data
Gaussian process model, Markov random field	Omics	Identify spatial domains from spatial transcriptomics
Graph neural network	Omics	Decipher cell-cell interaction
Fully supervised learning	Images	End-to-end prediciton model development
Self-supervised learning	Images	Learning a better representation for images
Multi-instance learning	Images	Integrate regional features for whole-slide image level prediction
Traditional machine learning	EHR	Integrate common clinical factors for prediction modeling
Large language model	EHR	Curate unstructure clinical notes

## 3 Multimodal integration

**FIGURE 1:**
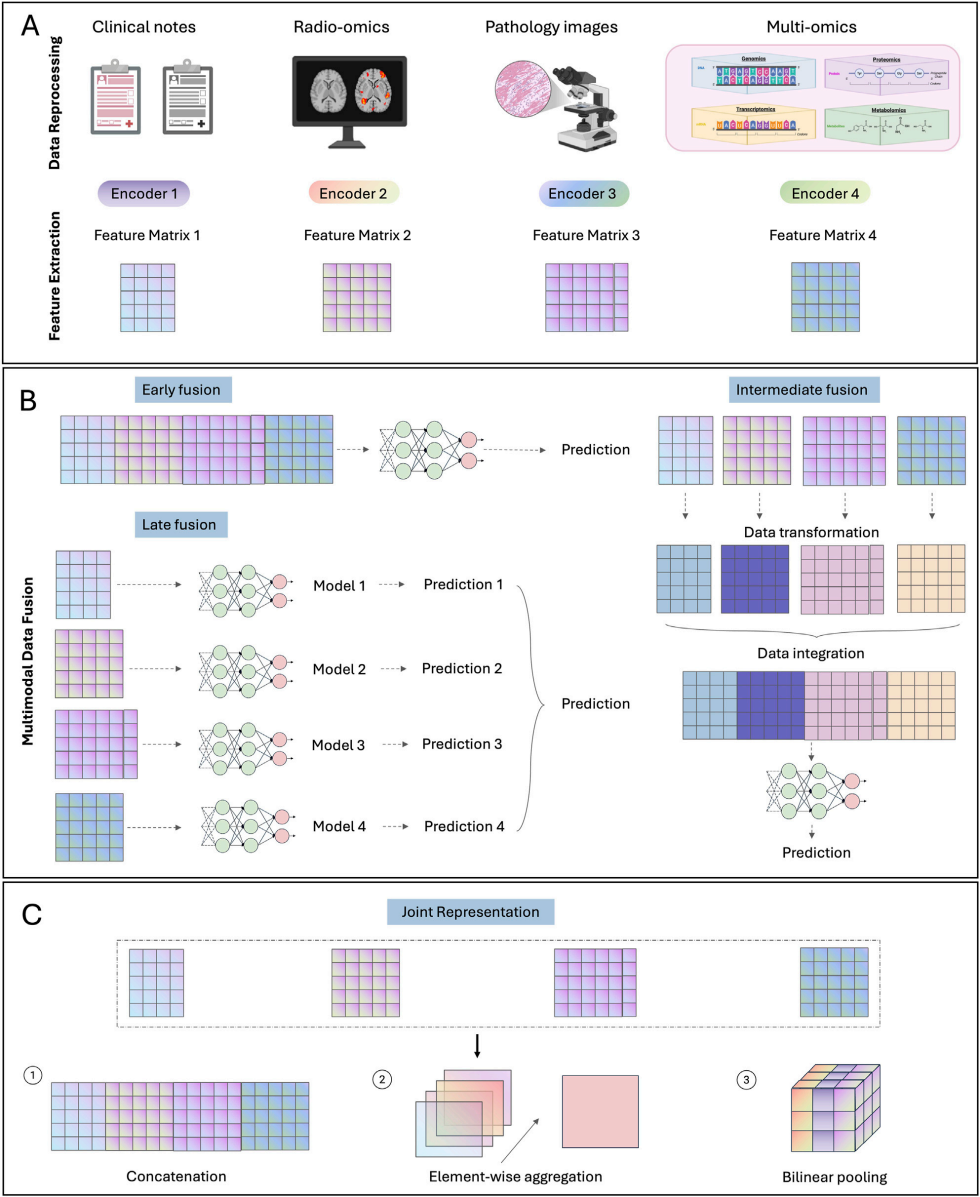
Multimodal fusion strategies. **(A)** Data preprocessing and feature extraction steps for common modalities. **(B)** Illustration of three multimodal fusion strategies: early, late and intermediate fusion. **(C)** Methods for generating joint representation.

Despite recent advancements in biomarker discovery and data processing techniques in each modality, patients exhibiting similar biomarkers can still have distinct prognoses and treatment responses (Kern, 2012). This deviation can potentially be attributed to the extensive heterogeneity inherent in cancer, where each modality captures only a fragment of the entire tumor profile, thereby hindering precise patient stratification. Integrating multimodal data can offer complementary insights across modalities, facilitating a more comprehensive evaluation of the tumor profile. Considering the complex and diverse data structures across different modalities, deep learning becomes an optimal approach due to its advantage in processing and aligning high-dimensional and complex data. Due to the innate complexity of different modalities and deep learning algorithms, most multimodal fusion strategies are still being explored in research settings. Based on the data fusion stages, multimodal fusion strategies can be divided into early, late and intermediate fusion strategies (Figures 1A,B).

### 3.1 Early fusion

Early fusion refers to integrating multimodal data into a unified feature matrix at an initial stage, followed by the development of a single model based on the integrated feature matrix for given prediction tasks. Typically, features from different data modalities are directly concatenated together into the integrated feature matrix. Due to the significant divergence between certain data modalities, such as images and gene expression matrices, pre-trained unimodal models are commonly employed to extract dimensionally aligned feature vectors for each independent modality (Stahlschmidt et al., 2022). Note that, although multiple models may be utilized during early fusion, they are fixed or slightly adjusted for feature extraction purpose without further extensive training. The main model for development during early fusion is the one utilizing the integrated feature matrix as input.

Since only one model is focused for extensive training during early fusion, this strategy tends to be simple to design and implement, as it alleviates the development of multiple individual models. However, it does not consider the crosstalk between different modalities and treat each modality uniformly, which may result in suboptimal performance when dealing with complex data modalities (Huang et al., 2020a). Moreover, since the model is trained on the integrated feature, early fusion is not well-suited for addressing missing data.

Early fusion has been widely applied for cancer survival prediction. This includes integration of similar data structures such as different omics data (Bichindaritz et al., 2021; Zhao et al., 2021) and clinical table curated from EHR data (Xie et al., 2019). Further studies have expanded to incorporate more distinct modalities such as histology images (Chen et al., 2022c; Mobadersany et al., 2018) and spatial cellular patterns within these images (Chen et al., 2022b). Another key application area is cancer subtyping. Previous studies have integrated histology images with omics or clinical data for molecular subtyping in breast cancer (Yang J. et al., 2022). In above studies, pretrained CNN models were utilized to extract deep features from images for multimodal alignment. Besides deep features, some studies used hand craft features from image modality during early fusion. For example, Hyun et al. curated several quantitate features (e.g., skewness, kurtosis and texture features) from radiomic images, and then combined them with clinical features for histological subtyping in lung cancer (Hyun et al., 2019).

### 3.2 Late fusion

In contrast to early fusion occurring at the input level, late fusion focuses on the decision level. Specifically, late fusion develops customized models for each modality to obtain a high-level summarization for decision making, usually a numeric score for given prediction tasks. These scores from independent modalities are then aggregated into a single score for the final prediction.

Since each modality is represented by a score through its specifically designed model, late fusion can handle missing modalities by majority vote or averaging unimodal scores (Ramanathan et al., 2022). As these scores are calculated specifically for each modality, late fusion also allows customized processing of complex data structures to enhance model performance. Furthermore, the use of numerical scores simplifies multimodal alignment by reducing the need for processing all modal input into aligned dimensions for integration. However, developing customized models typically requires additional computing resources and specific domain knowledge for each modality, which may increase the complexity for model implementation and deployment. In addition, similar to early fusion, late fusion does not account for potential crosstalk between different data modalities (Huang et al., 2020a).

Conventionally, late fusion integrates scores from each modality. Previous studies have calculated scores for image and multi-omics data respectively, and combined the scores with clinical features for biomedical applications such as diagnosis (Reda et al., 2018), recurrence prediction (Rabinovici-Cohen et al., 2022), and prognosis estimation (Sun et al., 2019; Wang H. et al., 2021). Besides score-based approaches, some studies employed feature selection strategy for multi-omics data to identify key gene expression levels or copy number values instead of relying solely on one score (Arya and Saha, 2021). For image modality, hand crafted features, including nuclei area and shape, have been commonly used (Shao et al., 2020). With the recent advancement in deep learning, deep features extracted from customized deep learning models have increasingly been adopted for the image modality (Liu et al., 2022; Vale-Silva and Rohr, 2021).

### 3.3 Intermediate fusion

Intermediate fusion represents a bridging stage between early and late fusion. It does not directly combine input data like early fusion nor customize specific models for each data modality as late fusion. Instead, it focuses on incorporating interaction between different modalities, which has not been addressed by early and late-stage fusion, to align multimodal data and generate improved low-level multimodal feature representation. This method backpropagates the loss to the input from each modality, thereby enabling a dynamic integration of multimodal signals tailored to the specific prediction task (Huang et al., 2020a). This design allows intermediate fusion to effectively model complex interactions across multiple modalities. However, due to its commonly hyper-parameterized nature, intermediate fusion is prone to overfitting the training data, thereby limiting its generalizability (Huang et al., 2020b).

To model interactions across modalities, similarity- and attention-based methods are most commonly used. Similarity-based methods assume different modal data from the same patients to be closer to each other in latent space compared to those from different patients. Therefore, the objective function is typically designed to maximize the similarity between different modalities from the same patient while minimizing the similarity for those from different patients. In practice, most studies adopt cosine similarity due to its scale-invariance and simplicity (Cheerla and Gevaert, 2019; Radford et al., 2021). There are also some studies calculating the mean square error (MSE) as the measure of similarity (Ding K. et al., 2023).

Attention-based method is inspired by the Visual Question Answering (VQA) that learns the association between words and objects from sentences and images respectively, the so-called attention (Fukui et al., 2016). The self-attention mechanism from the transformer structure has been widely used (Vaswani et al., 2023). It involves projecting the input into Q (query), K (key) and V (Value) vectors to learn attention between two modality embeddings. Then the attention-weighted embeddings from each modality can be concatenated together as the final multimodal representations. Previous studies have used the genomic data to query the key and the values from image data for calculating the co-attention between two modalities, enabling identification of image regions specifically related to given molecular aberrations (Chen et al., 2021c). Further studies refined this approach by incorporating the reverse direction: querying genomic data based on image data (Jaume et al., 2024; Zuo et al., 2022). Besides, several studies employed optimal transport (Xu and Chen, 2023) or hierarchical fusion (Li et al., 2022) to compute the attention (interaction) between modalities.

Concatenation is commonly used for fusing features from different modalities (Figure 1C) (Mobadersany et al., 2018). To include feature interactions, element-wise aggregation operators such as element-wise summation or multiplication (Hadamard product) have been employed. More sophistically, bilinear pooling (Kronecker product), which models pairwise interactions by calculating the outer product of two feature vectors, have been developed (Chen et al., 2022b; Chen et al., 2022c; Wang Z. et al., 2021). However, this operator usually results in a high-dimensional feature matrix. To reduce the high computational cost, factorized bilinear pooling methods have been developed based on low-rank matrix projections (Kim et al., 2017; Li et al., 2022a; Qiu et al., 2023).

Table 2 summarized above discussed studies. In practice, no fusion stage or operator consistently outperforms others across all scenarios. The choice of strategy should be guided by the specific data and prediction tasks.

**TABLE 2:** Summary of multimodal integration studies.

Study	Modalities	Fusion strategy	Cancer type	Sample size	Performance
Bichindaritz et al.	mRNA, DNA methylation	Early	BRCA	1097	c-index: 0.72
Zhao et al.	mutation, mRNA, methylation, CNV	Early	pan-cancer	3400	c-index: 0.69
Xie et al.	mutation, mRNA, protein, CNV, clinical	Early	pan-cancer	5748	c-index: 0.53–0.84
Chen et al.	WSI, mRNA, mutation, CNV	Early	pan-cancer	5401	c-index: 0.64
Mobadersany et al.	WSI, mutation, CNV	Early	LGG, GBM	769	c-index: 0.78
Chen et al.	WSI, mRNA, mutation, CNV	Early	LGG, GBM, KIRC	1186	c-index: 0.78
Yang et al.	WSI, clinical	Early	BRCA	123	AUC: 0.72
Hyun et al.	PET/CT, clinical	Early	lung	396	AUC: 0.86, ACC: 0.77, F1: 0.77. precision: 0.80, recall: 0.75
Reda et al.	MRI, clinical	Late	PRAD	18	ACC: 0.78
Rabinovici-Cohen et al.	MRI, clinical	Late	BRCA	1738	AUC: 0.75, specificity: 0.57, sensitivity: 0.90
Sun et al.	mRNA, CNV, clinical	Late	BRCA	1980	AUC: 0.85
Wang et al.	CT, mRNA	Late	lung	130	AUC: 0.73
Arya et al.	mRNA, CNV, clinical	Late	BRCA	1980	AUC: 0.95
Shao et al.	WSI, mRNA, CNV, methylation	Late	KIRC, KIRP, LUSC	787	c-index: 0.76, AUC: 0.78
Liu et al.	WSI, mRNA, CNV	Late	BRCA	1098	AUC: 0.94, ACC: 0.88
Vale-Silva et al.	WSI, clinical, mRNA, microRNA, methylation, CNV	Late	pan-cancer	11315	c-index: 0.79
Cheerla et al.	WSI, clinical, mRNA, microRNA, methylation	Intermediate	pan-cancer	11160	c-index: 0.78
Ding et al.	WSI, mRNA, CNV, methylation	Intermediate	COADREAD	571	c-index: 0.71
Chen et al.	WSI, mRNA, mutation, CNV	Intermediate	BLCA, BRCA, GBMLGG, LUAD, UCEC	3523	c-index: 0.65
Jaume et al.	WSI, mRNA	Intermediate	BLCA, BRCA, STAD, COADREAD, HNSC	2233	c-index: 0.63
Zuo et al.	WSI, mRNA	Intermediate	BRCA	427	c-index: 0.74, AUC: 0.75
Xu et al.	WSI, mRNA, mutation, CNV	Intermediate	BLCA, BRCA, GBMLGG, LUAD, UCEC	2831	c-index: 0.71
Li et al.	WSI, mRNA, CNV, clinical	Intermediate	BRCA	1015	c-index: 0.77, AUC: 0.81
Wang et al.	WSI, mRNA	Intermediate	BRCA	345	c-index: 0.72, AUC: 0.82
Qiu et al.	WSI, mRNA, mutation, CNV	Intermediate	BLCA, KIRC, KIRP, LUSC, LUAD, PAAD	2250	c-index: 0.68

## 4 Clinical application

**FIGURE 2:**
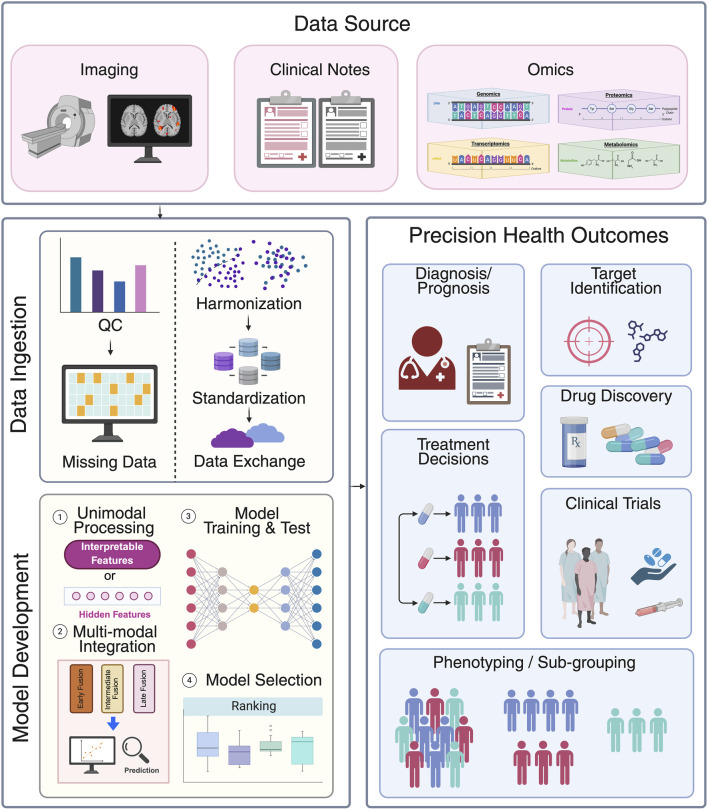
Data pipelines for multimodal data integration in clinical setting. First, various data modalities such as imaging, clinical notes and multi omics data are collected from data sources. Subsequentially, all data is ingested to data systems. This process involves quality control (e.g., presence of abnormal value or population prevalence), addressing missing data (e.g., discarding or imputing), harmonization (batch effects correction), standardization and loaded into systems for exchange. Model development involves four key steps: 1) unimodal processing: each data modality is first processed into interpretable features or hidden features via neural networks; 2) multi-modal integration: different data modalities are integrated together through early, intermediate, or late fusion strategy; 3) model training and test: different models are developed in curated datasets; and 4) model selection: The model with the best performance is selected to predict precision health outcomes such as diagnosis/prognosis, treatment decisions, patient sub-grouping, target identification, drug discovery, and to aid clinical trials. Created in BioRender. Wan, Z. (2025) https://BioRender.com/a7z643o.

In recent years, the rapid advancement of artificial intelligence (AI) and the explosive growth of multi-modal data have demonstrated remarkable potential of machine learning (ML) and deep learning (DL) in early cancer detection and diagnosis, molecular biomarker discovery and patient clinical outcome prediction (Figure 2). The following sections review key studies and recent progress for these three major application areas.

### 4.1 Early detection/diagnosis

Early detection and diagnosis of cancers can significantly improve patient survival rates, treatment effectiveness, and patient life quality. Traditional cancer diagnosis typically involves non-invasive imaging (e.g., radiological scans), followed by invasive biopsy taken for histological examination if suspicious regions of tissues are detected (Díaz et al., 2024). However, these approaches usually only rely on a single-modality screening, which may miss early-stage tumors (Jiang et al., 2024; Park et al., 2022). They also suffer from false positives and delayed image evaluation by time-constrained physicians (Weiss et al., 2017). AI-driven models can leverage diverse data modalities to uncover hidden patterns and to increase sensitivity and accuracy to detect pre-malignant changes before symptoms appear for some cancers. Iniyan et al. combined various imaging modalities (e.g., mammograms, ultrasound, and MRI) and proposed a technique by employing a fusion joint transfer learning for breast cancer diagnosis. The proposed method demonstrated superior performance than single-modality approaches by using histopathological images and ultrasound (Iniyan et al., 2024). A novel AI-based predictive system (AIPS) was developed to integrate radiological imaging, clinical information and genomics data to improve personalized risk stratification and classification accuracy in lung cancer. The AIPS models can achieve AUCs ranging from 0.587 to 0.910 in detecting the location of lung nodules in a cost-effective manner by reducing resource-intensive steps such as manual image annotation and complex feature engineering (Batra et al., 2024).

Recent advancement in single-cell and spatial omics have revolutionized multi-modal data integration, enabling mapping of cell-specific gene expressions with the incorporation of spatial location information and associating with other traditional data modalities (Chen S. et al., 2024). Interestingly, Bae et al., developed a deep learning framework SPADE to identify important genes associated with morphological contexts by combining spatial transcriptomics data with overlapping high-resolution images. Both spatial gene expression and H&E imaging data were fed into a VGG-16 convolutional neural network (CNN) to extract imaging features based on spatial coordinates. The DL framework was applied for malignant vs. benign tissue classification and yielded an accuracy of 0.91, while the single-data modality with the accuracy dropping to 0.60 without imaging data (Bae et al., 2021).

The AI models with enhanced accuracy can alleviate the workload of pathologists for manual microscopic inspection and accelerating early detection of cancers, particularly in resource-limited settings.

### 4.2 Molecular biomarker discovery

Biomarkers play a crucial role in cancer risk stratification and personalized treatment design (Naik et al., 2020). The well-established molecular biomarkers include oncogenic mutations (e.g., TP53, BRAF V600 E, MYC amplification, etc.), therapeutic biomarkers (e.g., TMB, PD-L1, MSI status, tumor-infiltrating lymphocytes, etc.) as well as some emerging biomarkers (e.g., ctDNA, DNA methylation markers, etc.). The main advantage of multimodal data integration in biomarker discovery is to capture tumor complexity across different biological layers and to reveal shared associations across molecular alterations, tissue morphology and clinical attributes, aiming to provide a comprehensive molecular and phenotypic landscape of tumors (Lipkova et al., 2022; Llinas-Bertran et al., 2025; Yang et al., 2025).

One of the most successful multi-modal data integration applications is to fully leverage the easy accessibility of imaging data (e.g., H&E, CT scans, X-ray, MRI, etc.) in clinical setting on biomarker identification and prediction (Chiu and Yen, 2023; Mi et al., 2024; Pang et al., 2022). Unlike genomics sequencing or multi-omics profiling that requires specialized instruments and process as well as its high cost, imaging data is readily available and non-invasive in standardized cancer patient diagnosis and treatment clinical practice, making it a perfectly complementary resource to molecular data for biomarker discovery. The technical development of ML and DL further make imaging data as an efficiently and widely adopted tool by extracting quantitative morphological features to correlate with available patient molecular and clinical data.

One of the seminal studies by Coudray et al. (2018), demonstrated that H&E-stained WSIs can be used for lung cancer histological group classification and directly infer the most common mutations by a deep convolutional neural network (inception v3). The predictive models achieved promising performance with AUC from 0.73 to 0.85 for prediction of six lung cancer driver mutations. This study opened a new opportunity for integrating massive clinical imaging data into biomarker prediction, especially in the absence of omics data. More similar studies were followed for mutation prediction by imaging data for different cancer types (Bilal et al., 2021; Chen M. et al., 2020; Jang et al., 2020; Loeffler et al., 2022). More recently, some studies (Fu et al., 2020; Kather et al., 2020) attempted to predict any clinically actionable genetic alterations in pan-cancers by using different approaches, such as weakly supervised learning.

Besides mutations, AI technologies have also been applied to predict gene expression by using imaging data. Schmauch et al. (2020). Developed a novel deep learning algorithm HE2RNA by training TCGA datasets to predict mRNA expression directly from whole-slide histology images without the need for expert annotation. This method also provided visual spatialization of gene expression and it was validated by CD3- and CD20-stained samples. Moreover, this application can be expanded to spatial level. James Zou’s lab used matched spatial transcriptomics data and H&E-stained histopathology images from breast cancer patients, enabling the prediction of spatial gene expression map directly from standard imaging slides (He B. et al., 2020). The developed DL algorithm ST-Net demonstrates robust generalization capability to other datasets, offering a cost-effective alternative to spatial transcriptomics.

Additional biomarkers such as TMB (Huang et al., 2020a; Jain and Massoud, 2020; Niu et al., 2022), MSI status (Gustav et al., 2024; Kather et al., 2019; Lee et al., 2021), PD-L1 (Li et al., 2024; Liang et al., 2024; Shamai et al., 2022), hormone-receptor status (Naik et al., 2020; Wu et al., 2024) have also been successfully predicted by imaging data leveraging AI technologies. These identified associated morphological features can be served as non-invasive biomarker surrogates when lacking extensive molecular profiling to guide patient tailored treatment strategy.

### 4.3 Prediction of patient prognosis and treatment response

Cancer patient prognosis and treatment response are usually only assessed by clinical variables; however, this trend is changing towards integrating multi-modal data to this end. The additional layers of information can provide a more comprehensive picture of underlying characteristics affecting patient survival and treatment response as well as the hidden relationships between these features. The estimate of patient clinical outcome has become crucial for physicians to monitor patient disease progression and to design effective therapeutic strategy.

Huang et al. (2019) introduced a deep learning framework called SALMON (survival analysis learning with multi-omics neural network) to incorporate diverse data types, including multi-omics and clinical information such as age and hormone receptor status. To solve the high dimensionality issue inherent in omics data, the authors first constructed co-expression networks to identify gene modules as eigengenes and investigate the contributions of these gene modules to the hazard ratio. This approach successfully reduced the feature space by approximately 99% and largely increased model robustness and effectiveness, leading to enhanced survival prognosis prediction for breast cancer patients. In addition, the gene modules identified as most significantly associated with the hazard ratio were further evaluated with pathway enrichment analysis to elucidate gene regulation mechanisms to enhance biological interpretation. Other similar studies using DL algorithms for patient prognosis prediction have been published in other cancer types (Boehm et al., 2022; Chaudhary et al., 2018; Steyaert et al., 2023).

The multi-modal integration can also be applied to predict treatment response by utilizing clinical trial data (Schweinar et al., 2024). This important study (Esteva et al., 2022) leveraged five phase III randomized trails, encompassing more than 5,000 prostate cancer patients with a median follow-up of 11.4 years. The team developed a multimodal deep learning model to predict long-term clinical outcome and achieved a 9.2%–14.6% relative improvement compared to risk stratification tools. Furthermore, this study integrated imaging data with clinical information, and provided pathologist interpretations on identified tissue clusters, demonstrating one of the main merits of multi-modal data integration than unimodal models–increased biological interpretation and clinically relevant inference.

The application has been expanded to the prediction of other clinical outcome such as recurrence (Lee et al., 2020; Yamamoto et al., 2019), drug side effects and toxicity (Men et al., 2019; Mukherjee et al., 2025) by incorporating diverse data modalities, all of which have significantly improved patient survival rates and optimized treatment strategy in precision oncology.

Moreover, the application is not only at the research setting, it has been also integrated into multidisciplinary tumor boards (MTBs) through the lens of clinicians—surgeons, medical oncologists, and radiation oncologists—emphasizing the clinically transformative potential (Nardone et al., 2024). Surgeons view AI as a tool for enhancing intraoperative decision-making and surgical education, with models like GoNoGoNet (Laplante et al., 2023) and DeepCVS (Mascagni et al., 2022) offering real-time anatomical guidance and safety assessments during procedures. Medical oncologists leverage AI for molecular profiling, treatment selection, and clinical trial optimization, with platforms such as Watson for Oncology and radiogenomic models predicting treatment responses and genetic mutations (Lee and Lee, 2020). Radiation oncologists benefit from AI in treatment planning and toxicity prediction, using tools like the Radiation Planning Assistant (RPA) to automate contouring and dose optimization (Court et al., 2018).

Interestingly, virtual technologies also play transformative roles in enhancing cancer diagnosis, patient treatment and support. For example, Z˙ydowicz et al. introduced how 3D printing and augmented reality (AR)/virtual reality (VR) can improve surgical precision, reduce reoperation rates, and support rehabilitation and training (Żydowicz et al., 2024). Metaverse is an AI-integrated platform for immersive surgical planning and education, showing broader potential in healthcare (Żydowicz et al., 2024). These emerging AI tools require further clinical validation, ethical safeguards, and accessibility improvements to ensure safe and collaborative integration into existing clinical practices.

## 5 Challenges

### 5.1 Missing data

A major challenge in developing multimodal deep learning models for clinical application is dealing with missing data. For instance, molecular features might be missing for certain patients, as not all patients undergo genomic tests. Additionally, curating structured data from clinical notes is complicated, possibly leading to missing clinical variables. Survival data may also be missing or inaccurate when follow-up periods are limited. In cases where an entire modality (e.g., H&E slides) is often missing in certain patients, discarding the modality data for those patients can be an option (Jun-Ho and Lee, 2019). This approach is more compatible with late fusion since it combines scores from each modality, and the combination process is typically robust, regardless of the number of input scores. Conversely, if only a certain part of a modality (e.g., clinical variables) is missing, imputation methods can be used to estimate the missing values based on the available data (Luo Y. 2022; Yoon et al., 2019).

### 5.2 Multimodal alignment

Since different modalities can exhibit distinct dimensionalities, numerical scales, and data structures, it is necessary to align them into compatible formats before integration. With recent advances in vision-language models, the transformer architecture has become the paradigm for processing both image and language modalities into numerical representation vectors (Bordes et al., 2024). However, for molecular and structured clinical data with tabular formats, deep learning methods encounter significant challenges due to the highly different distributions across feature values (Grinsztajn et al., 2022). Traditional normalization methods are ineffective here, as gradient descent optimization and dropout regularization can disrupt this normalization, leading to instability in training, especially for deep neural networks (Grinsztajn et al., 2022). To address this, self-normalizing neural networks (SNNs) have been introduced to preserve the data distribution at each layer (Klambauer et al., 2017). Further research has also incorporated biological knowledge into SNNs to enhance model performance (Jaume et al., 2024).

### 5.3 Insufficient interpretability

Despite impressive performance, deep learning models are challenging to interpret due to their hyper-parameterized structures. Previous studies have introduced several *post hoc* interpretation strategies for different modalities. For image modality, attention-mechanisms (Li et al., 2021a; Shao Z. et al., 2021) are used to identify tiles in whole slide images that significantly contribute to the model’s output, with class activation maps (Afify et al., 2023; Selvaraju et al., 2017) employed to highlight important regions within each tile. For molecular data, methods based on Shapley Additive Explanation (SHAP) (Singh et al., 2017; Chen et al., 2022c) are utilized to determine the importance on each gene or pathway level. However, these interpretations remain abstract and are insufficient for drawing precise biological insights in clinical settings. Furthermore, in a multimodal context, interpretation becomes more complicated due to the necessity of disentangling the contributions from different modalities.

### 5.4 Data interoperability

Different institutions often maintain their own IT infrastructures for storing patient data, resulting in varied syntactic structures among different sources, which complicates efforts to centralize data. Furthermore, different workflows for curating these datasets introduce significant semantic variability across sources. As clinical guidelines are keep evolving, several clinical practice such as definition of cancer stage and standard of care can vary over time, hindering analysis of retrospective data. To support data sharing and centralization, several initiatives, including The Cancer Genome Atlas (TCGA), the Genomic Data Commons (GDC), the Database of Genotypes and Phenotypes (dbGAP), American Association for Cancer Research project Genomics Evidence Neoplasia Information Exchange (AACR project GENIE), The Cancer Imaging Archive (TCIA), the European Genome-phenome Archive (EGA), the Genomics Pathology Imaging Collection (GPIC), the Clinical Proteomic Tumor Analysis Consortium (CPTAC) are working towards standardizing data sources into a uniform structure (Weinstein et al., 2013; Zhang Z. et al., 2021; Tryka et al., n.d.; The AACR Project GENIE Consortium et al., 2017; Clark et al., 2013; Lappalainen et al., 2015; Jennings et al., 2022; Li Y. et al., 2023).

Another challenge for data sharing is the diversity of institutional privacy policies. Researchers must often undergo a series of legal reviews to comply with each institution’s data-sharing policies, which can strongly delay the sharing process and hampers model development. Federated learning offers an alternative solution. This approach enables model development through distributed and decentralized systems, allowing institutions to comply with privacy laws and regulations without sharing confidential data directly (Rieke et al., 2020).

### 5.5 Clinician acceptance

Currently, one of the key challenges in the application of AI on multi-modal data from clinical perspective is clinician acceptance. Because of the “black box” nature of many multi-modal AI models described above, the interpretability and transparency of AI models have become the main concerns from clinicians, especially when they cannot fully understand or validate against their own expertise and experience. Additionally, discrepancies between AI-driven insights and established clinical workflows can create friction, particularly if the system’s outputs are not seamlessly integrated into existing decision-making processes. Despite the rapid advancement of AI technologies, there remains gaps between their capabilities and alignment with clinical reasoning. Building clinician trust requires not only technical robustness but also thoughtful model design that prioritizes explainability, usability, and clinical translatability.

### 5.6 Generalizability of AI insights

Another significant limitation in applying AI to multi-modal data lies in the generalizability of the obtained AI results across diverse clinical settings and patient populations. Multi-modal models often rely on data from specific cohorts or geographic locations, which may not capture the full spectrum of variability in clinical practice, demographics, or disease presentation. As a result, models trained on one dataset may perform poorly when deployed in different environments, leading to difficulties of generalization of the obtained prediction results and AI insights. This issue is further compounded by the complexity of aligning and harmonizing heterogeneous data types—such as imaging, genomics, and electronic health records—which may be collected using different protocols or standards. Rigorous external validation and thorough consideration of data provenance are essential to overcoming key barriers to the widespread adoption of AI in real-world clinical workflows.

## 6 Discussion and outlook

### 6.1 Longitudinal multimodal fusion

Cancer is a dynamic process that evolves over time, driven by an intricate interplay of genetic, environment, and phenotypic changes. Most current cancer research is cross-sectional studies, which capture only a single snapshot of cancer at a specific timepoint (Avancini et al., 2024; Harle et al., 2020). In contrast, longitudinal data provides a more comprehensive perspective with patient temporal information across different disease phases, enabling the tracking of cancer progression and monitoring of treatment response (Zhuang et al., 2025). Harnessing AI-driven approaches to model longitudinal, multi-modal data presents a promising opportunity to increase predictive performance in cancer diagnosis, prognosis and clinical outcome. Some pioneering studies have explored various deep learning methods—such as recurrent neural network (RNN), transformers, self-supervised learning (SSL), reinforcement learning (RL) —by incorporating time embedding to analyze longitudinal data. However, most of these studies have focused on single-modality data, such as medical imaging (Gao et al., 2024; Gao et al., 2025; Shen et al., 2023) or molecular profiling (Ballard et al., 2024; Zhang et al., 2024; Wekesa and Kimwele, 2023), without fully incorporating the wealth of information available across multiple modalities. This leaves significant untapped opportunity to leverage the strength of different data modalities for a more holistic and temporally resolved characterization of tumors (Zhuang et al., 2025).

Despite these advances, a major gap remains between current AI models and an ideal longitudinal fusion system. Most existing approaches primarily focus on relatively controlled datasets but struggle to align and integrate heterogeneous data types collected at irregular intervals—common in clinical practice—while also lack robust algorithm to handle missing data effectively or capture complex temporal dependencies across modalities (de Mortanges et al., 2024; Loni et al., 2025). An ideal system would seamlessly model time-aware interactions between different data modalities, dynamically adapting to continuously evolving patient status and treatment responses over time. Bridging this gap will require new analytical frameworks that are both modality-agnostic and temporally flexible, capable of learning robust representations from incomplete and asynchronous data streams.

Besides the methodologies, progresses in this area is also constrained by limited available public longitudinal, multimodal cancer datasets. The Cancer Genome Atlas (TCGA) and Clinical Proteomic Tumor Atlas Analysis Consortium (CPTAC) provide longitudinal clinical follow-up and survival outcomes, although molecular profiling is primarily cross-sectional. The National Lung Screening Trial (NLST) (National Lung Screening Trial Research Team, 2011) includes serial CT imaging and clinical metadata over multiple timepoints. These emerging resources offer solid foundation and partial solution. Developing well-annotated, longitudinal, multimodal datasets will be critical to enable reproducible research and advancing next-generation AI in oncology.

### 6.2 Integrate single-cell and spatial omics data

Compared to bulk-level omics data, single-cell and spatial omics data enable molecular characterization of individual cells within a population, providing deeper insights into complex cellular system in their spatial context (Du et al., 2024; Lee et al., 2024). Single-cell and spatial omics data have been unprecedentedly accumulated due to the rapid advancement of state-of-the-art sequencing technologies over the past decades. With the surge of AI/ML techniques—coupled with different fusion strategies described in this review—there is growing confidence in the potential of applying advanced AI/ML algorithms on these high-resolution data (Halawani et al., 2023). Such approaches have the power to enhance predictive performance and reveal novel biological insights. However, some challenges still remain (Athaya et al., 2023), for example, a lack of standardized workflow for data selection, preprocessing, normalization, and harmonization poses a significant barrier (Fan et al., 2020). In addition, the design of DL architecture needs to consider the high-dimensionality, sparsity and noise inherent in these data types (Stegle et al., 2015). Furthermore, integrating omics datasets across bulk, single cell and spatial levels with other data modalities remain a crucial challenge that must be addressed in future work to promote comprehensive cancer research.

### 6.3 Foundation models in healthcare

Foundation models are large generalist models pre-trained on extensive datasets to learn representations from vast amounts of information (Bommasani et al., 2022). They can be easily adapted to specific tasks through fine-tuning, often outperforming specialist models developed *via* fully supervised manner. These models show significant promise across various healthcare data modalities, including clinical notes, imaging data, and sequencing data. For instance, large language models like ChatCAD, MedAgents, and Med-PaLM can summarize clinical notes into reports and serve as virtual healthcare consultants (Tang J. et al., 2025; Tang et al., 2024; Singhal et al., 2023). Vision foundation models have been adapted for clinical tasks such as tissue segmentation, lesion detection, and survival prediction (Wang et al., 2022b; Chen R. J. et al., 2024; Xu Hang et al., 2024). Regarding sequencing data, the DNA foundation model Evo has been proposed to estimate gene essentiality (Nguyen et al., 2024). Protein language models like AlphaFold2/3, ESM3, and ProGen have been developed for predicting protein structure and function (Jumper et al., 2021; Abramson et al., 2024; Hayes et al., 2024; Madani et al., 2023). Foundation models like scVI, scGPT, and Geneformer have been utilized for cell type annotation and correcting batch effects in single-cell datasets (Lopez et al., 2018; Cui et al., 2024; Theodoris et al., 2023). By integrating data from different modalities, multimodal foundation models enable cross-modality applications and enhance performance beyond single-modality limitations. Vision-language models such as PathChat, LLaVA-Med, Clinical-BERT, and XrayGPT can generate clinical reports and provide insights based on clinical images (Lu et al., 2024b; Li C. et al., 2023; Huang Shih-Cheng et al., 2020; Thawakar et al., 2025). Among them, PathChat has received FDA Breakthrough Device Designation (PathChat, 2025). Molecular language models like BioMedGPT can analyze sequencing datasets given natural language queries (Zhang et al., 2024).

Despite the rapid advancements and promising potential of foundation models, several challenges persist. Training these models typically requires enormous amounts of data, which is often scarce in clinical settings due to difficulties in data curation and related ethics and privacy regulations (Bommasani et al., 2022; Willemink et al., 2020). The large architecture of these models and the extensive data requirements also pose challenges to computing infrastructures (Ding N. et al., 2023; Touvron et al., 2023). Additionally, due to the lack of grounding and potential biases in training data, AI models may generate fatally inaccurate results, the so-called AI hallucinations, which can be particularly dangerous when incorrect information is provided to patients without sufficient medical knowledge (Maleki et al., 2024). Thus, fostering data standardization, reducing computational costs, and ensuring safety controls are critical for the future development of foundation models.

### 6.4 Multimodal fusion strategy selection

Three strategies for multimodal fusion are discussed above, namely, early, late, and intermediate fusion (Huang Shih-Cheng et al., 2020). Each strategy possesses distinct strengths and weaknesses, and no single strategy is universally optimal for all scenarios. Early fusion merges input features directly, making it simple to implement; however, it cannot handle missing modalities in the input data (Baltrusaitis et al., 2019). Conversely, late fusion customizes separate models to generate predictions for each available modality, addressing the missing modalities by averaging predictions. Despite its advantages, late fusion requires substantial computational costs for developing and implementing independent models (Baltrusaitis et al., 2019). Both early and late fusion strategies overlook the interactions between different modalities. Intermediate fusion tackles this issue by incorporating architectures that model inter-modality interactions, thereby extracting orthogonal features from each modality for improved prediction performance (Huang et al., 2020a). Nonetheless, this approach introduces additional parameters, making the model prong to overfit. Therefore, when deciding the fusion strategy, various factors should be considered, such as prediction task, computational resources, sample size, and proportion of missing values.

## Glossary

AI: Artificial intelligence
EHR: Electronic health record
MRI: Magnetic resonance imaging
CT: Computed tomography
TCGA: The Cancer Genome Atlas
PCA: Principal component analysis
KEGG: Kyoto Encyclopedia of Genes and Genomes
H&E: Hematoxylin and eosin
WSI: Whole-slide image
DL: Deep learning
SSL: Self-supervised learning
MLP: Multilayer perceptron
BYOL: Bootstrap Your Own Latent
DINO: Distillation with No Labels
iBOT: Image BERT Pre-Training with Online Tokenizer
MIL: Multi-instance learning
ViT: Vision Transformer
HIPT: Hierarchical Image Pyramid Transformer
CNN: Convolutional neural networks
NLP: Natural language processing
MSE: Mean square error
VQA: Visual Question Answering
ML: Machine learning
AIPS: AI-based predictive system
TMB: Tumor mutation burden
MSI: Microsatellite instable
AUC: Area under the curve
SALMON: survival analysis learning with multi-omics neural network
MTB: multidisciplinary tumor boards
RPA: Radiation Planning Assistant
AR: Augmented reality
VR: Virtual reality
SNN: self-normalizing neural network
SHAP: Shapley Additive Explanation
GDC: Genomic Data Commons
dbGAP: Database of Genotypes and Phenotypes
AACR project GENIE: American Association for Cancer Research project Genomics Evidence Neoplasia Information Exchange
TCIA: The Cancer Imaging Archive
EGA: European Genome-phenome Archive
GPIC: Genomics Pathology Imaging Collection
CPTAC: Clinical Proteomic Tumor Analysis Consortium
RNN: Recurrent neural network
RL: Reinforcement learning
NLST: National Lung Screening Trial
LGG: Brain Lower Grade Glioma
BRCA: Breast invasive carcinoma
COAD: Colon adenocarcinoma
GBM: Glioblastoma
BLCA: Bladder Urothelial Carcinoma
KIRC: Kidney renal clear cell carcinoma
KIRP: Kidney renal papillary cell carcinoma
LUAD: Lung adenocarcinoma
LUSC: Lung squamous cell carcinoma
PRAD: Prostate adenocarcinoma
READ: Rectum adenocarcinoma
STAD: Stomach adenocarcinoma
UCEC: Uterine Corpus Endometrial Carcinoma
PAAD: Pancreatic adenocarcinoma
HNSC: Head and Neck squamous cell carcinoma.
